# Rapid cost-effectiveness analysis: hemodialysis versus peritoneal dialysis for patients with acute kidney injury in Rwanda

**DOI:** 10.1186/s12962-024-00545-0

**Published:** 2024-04-30

**Authors:** Cassandra Nemzoff, Nurilign Ahmed, Tolulope Olufiranye, Grace Igiraneza, Ina Kalisa, Sukrit Chadha, Solange Hakiba, Alexis Rulisa, Matiko Riro, Kalipso Chalkidou, Francis Ruiz

**Affiliations:** 1 London School of Hygiene and Tropical Medicine, London, UK; 2 Center for Global Development, International Decision Support Initiative, iDSI, London, UK; 3 Rwanda Social Security Board, Kigali, Rwanda; 4 Clinton Health Access Initiative, Kigali, Rwanda; 5 The University Teaching Hospital of Kigali, Kigali, Rwanda; 6 World Health Organization, Kigali, Rwanda; 7 Imperial College London, London, UK

**Keywords:** Rwanda, Rapid cost-effectiveness analysis, Economic evaluation, Adaptive health technology assessment, Health technology assessment, Dialysis, Acute kidney injury

## Abstract

**Background:**

To ensure the long-term sustainability of its Community-Based Health Insurance scheme, the Government of Rwanda is working on using Health Technology Assessment (HTA) to prioritize its resources for health. The objectives of the study were to rapidly assess (1) the cost-effectiveness and (2) the budget impact of providing PD versus HD for patients with acute kidney injury (AKI) in the tertiary care setting in Rwanda.

**Methods:**

A rapid cost-effectiveness analysis for patients with AKI was conducted to support prioritization. An ‘adaptive’ HTA approach was undertaken by adjusting the international Decision Support Initiative reference case for time and data constraints. Available local and international data were used to analyze the cost-effectiveness and budget impact of peritoneal dialysis (PD) compared with hemodialysis (HD) in the tertiary hospital setting.

**Results:**

The analysis found that HD was slightly more effective and slightly more expensive in the payer perspective for most patients with AKI (aged 15–49). HD appeared to be cost-effective when only comparing these two dialysis strategies with an incremental cost-effectiveness ratio of 378,174 Rwandan francs (RWF) or 367 United States dollars (US$), at a threshold of 0.5 × gross domestic product per capita (RWF 444,074 or US$431). Sensitivity analysis found that reducing the cost of HD kits would make HD even more cost-effective. Uncertainty regarding PD costs remains.

Budget impact analysis demonstrated that reducing the cost of the biggest cost driver, HD kits, could produce significantly more savings in five years than switching to PD. Thus, price negotiations could significantly improve the efficiency of HD provision.

**Conclusion:**

Dialysis is costly and covered by insurance in many countries for the financial protection of patients. This analysis enabled policymakers to make evidence-based decisions to improve the efficiency of dialysis provision.

## Introduction

### Background

Rwanda’s community-based health insurance (CBHI) scheme covers more than 80% of the population, most of whom are in the informal sector [1]. The scheme has three main funding sources: member contributions, government subsidies, and donors, and operates mostly on a fee-for-service basis [2]. Members are entitled to a comprehensive benefits package covering drugs and medical services, and their contributions vary based on income level [3]. Co-payments are 200 Rwandan francs (RWF) at the health post level, and 10% of the total bill at higher levels of care [3]. As part of the scheme’s success, the government continues to face growing demand for a wide range of healthcare services, which it must balance with an estimated $58 per capita expenditure on health [4].

To strengthen the financial sustainability of the CBHI scheme, health technology assessment (HTA) is being introduced to support explicit, evidence-informed priority setting [5]. As a first step, a rapid cost-effectiveness analysis on dialysis for acute kidney injury (AKI) was undertaken [6].

Dialysis is a common topic of interest for low- and middle-income countries (LMICs) facing a growing burden of non-communicable diseases due to its high costs [7]. Across LMICs, only 2–5% of patients needing treatment receive it; for many, it is unaffordable [8]. At the time of analysis, six weeks of dialysis was covered by CBHI in Rwanda with a 10% co-pay, averaging 218,000 RWF out of pocket per patient [9]. For scale, this represents 25% of GDP per capita [10].

Some LMICs have conducted cost-effectiveness analyses on dialysis to inform their coverage decisions [11, 12]. However, these have been disproportionately focused on dialysis for patients with end-stage renal disease (ESRD) [13–16]. ESRD is the last stage of chronic kidney disease (CKD), which permanently impairs kidney function and renders patients ‘dialysis dependent’ to survive.

Dialysis is also used to treat AKI, which, unlike ESRD, temporarily impairs kidney function. AKI is reversible if diagnosed and treated early. Depending on the severity of a kidney injury, patients may only need dialysis for a limited period to allow for at least partial, and sometimes full recovery of kidney function.

LMICs bear a disproportionate amount of the globally estimated burden of AKI [17], and in these countries, it is commonly a disease of the young, often caused by a single, curable condition [17–20]. In Rwanda, the most common comorbidities include malaria; pneumonia; sepsis; pregnancy-related conditions such as eclampsia and hypertension; intoxication caused by treatment from traditional healers; and diabetes [21]. The median age for AKI patients in Rwanda is 38 years old, and the mortality rate is 34% [21]. However, barriers to optimal management of AKI in Rwanda remain. These include knowledge gaps among healthcare providers, sub-optimal diagnostic capacity, particularly in sub-tertiary hospitals, and limited treatment options [22].

The main treatment options for AKI in Rwanda are hemodialysis (HD) and peritoneal dialysis (PD). Evidence suggests little difference when comparing HD and PD in terms of their clinical outcomes or the risk of complications – though the evidence base remains moderate to poor [23].

Currently, all dialysis provision in Rwanda is exclusively delivered in the hospital setting. Most of this provision is HD, with a small proportion being PD. While in other settings, HD is often provided in hospitals and PD in smaller facilities or at home this type of PD was discontinued a few years ago in Rwanda. This was partially because of challenges in sourcing dialysate and difficulty in guaranteeing hygienic conditions for at-home PD.

### Aim and objectives

At the time of writing, Rwanda’s CBHI benefits package officially covered up to six weeks of dialysis per patient with AKI. Dialysis for ESRD was not covered. However, the diagnosis of AKI versus CKD can sometimes be challenging, especially when there is a previously undiagnosed kidney dysfunction. Due to the considerable cost of providing dialysis, this study aimed to help the Rwandan CBHI scheme decide on the optimal delivery of dialysis services.

The objectives of the study were to rapidly assess (1) the cost-effectiveness and (2) the budget impact of providing PD versus HD for patients with AKI in the tertiary care setting in Rwanda. This may be the first study of its kind comparing dialysis modalities for AKI in LMICs where both HD and PD are provided exclusively at the tertiary care level.

## Methods

Our rapid cost-effectiveness analysis used an ‘adaptive’ HTA (aHTA) approach, which adjusts HTA methods for time, data, and capacity constraints [24]. To respond to policy makers’ demand, the aim was to complete the assessment in six weeks. It was thus decided to use a rapid cost-effectiveness analysis which builds basic economic models using opportunistically or rapidly sourced local data [24]. The assessment used the international decision support initiative (iDSI) reference case for economic evaluation as a guide [6, 25]. Table 1 summarizes the application of the eleven iDSI reference case principles. Highlighted rows indicate principles that were adapted for time and data constraints.

**Table 1:** Methodological approach using the iDSI reference case

Principle	The analysis should …	Dialysis approach
Transparency	Be clearly communicated	A ‘learn-by-doing’ approach was undertaken to ensure stakeholder engagement, learning, and translation of results
Comparators	Reflect decision problem. ‘No comparator’ optional	The comparator was HD, to reflect the decision problem and local standard of care. ‘No comparator’ was excluded as it was not considered a reasonable policy option
Evidence	Consider all available evidence	Dialysis was partially selected due to already available local cost data, and a recent systematic review on clinical effectiveness. Additional data needs were supplemented by rapid review and personal communication with co-authors
Health outcomes	Be appropriate to decision problem, capture positive and negative effects on length and quality of life, and be generalizable across disease states	Quality-adjusted life years (QALYs) were selected given availability of evidence from other jurisdictions
Costs	Reflect all differences in intervention and comparator costs	Costs reflect best available evidence on HD and PD, though limitations with PD data affect the certainty of results. No estimation of changes due to (diseconomies) of scale were made
Time horizon	Be sufficient to capture all costs and effects	A lifetime horizon was used
Non-health effects and costs outside the health budget	Be identified if relevant to the research question	The analysis’ focus was limited to the payer perspective, due to lack of locally available data to inform the optional societal perspective
Heterogeneity	Explore sub-populations	Two age groups were explored – 15–49 and over 50 to reflect the young age of most patients undergoing dialysis in Rwanda
Uncertainty	Be appropriately characterized	Deterministic and probabilistic sensitivity analysis were undertaken
Constraints	Evaluation budget impact including infrastructural/ resource constraints	Budget impact analysis was undertaken and is available in Supplement 1. Infrastructural constraints were likely underestimated for PD due to limited data
Equity considerations	Consider equity implications	Equity implications were considered only qualitatively

### Population and subgroups

The population for this analysis were dialysis-eligible patients with AKI in a tertiary care facility in Rwanda. Two age groups were considered: patients aged 15–49 and patients aged 50 and above.

### Comparators

The intervention was tertiary care delivered PD, compared with tertiary care delivered HD. In the base case, the model assumed that patients receive the maximum allotted care covered by CBHI. This included three sessions per week for six weeks of HD or six weeks of continuous ambulatory PD for hospitalized patients.

### Model structure and assumptions

A de novo Markov model was used to reflect the costs and effects of the initial acute condition of AKI combined with the long-term health effects that can follow the condition. The cycle length was one year (Appendix 1). The model was informed by the published literature and validated through consultation with local and international nephrology specialists. Patients enter the model at the tertiary care facility, starting on hospital HD or hospital PD. Over time, patients may stay with the same modality or switch modalities. They may develop complications or not, and subsequently fully recover rendering them dialysis independent or partially recover with no further treatment. They may then die from AKI progressing to CKD, from co-morbidities, or from natural causes.

### Modelling perspective and scenarios

A payer perspective was used. The payer perspective included all direct medical costs to Rwanda Social Security Board (RSSB) plus salaries, overhead, and depreciation of the HD machines paid by the Ministry of Health. A ‘decreased provision’ scenario was also explored, which assumed the actual number of sessions patients receive on average was five instead of the full eighteen sessions [9].

### Evidence for model parameters

Given an initially short timeline, a pragmatic approach was taken to select data to inform the model. Available local data was supplemented by a rapid literature search and sources known to the authors (Appendix 2). Where needed, gaps were addressed based on personal communication involving Rwandan nephrologists and international experts.

### Cost and resource use

Costs and resource use data were sourced primarily from a 2018 RSSB Utilization and Expenditure Review on Dialysis made available by RSSB [9]. These were supplemented by published data from other jurisdictions and assumptions made by co-authors on this study. The total cost for HD, PD, and palliative care are expressed as per patient unit costs and reflect the cost of providing HD and PD at the tertiary care level (Table 2). Direct medical costs (including catheters, drugs, lab tests, kits, dialysate, other consumables, and palliative care) were sourced from an average across four facilities for HD and one facility for PD. Direct non-medical costs (costs of healthcare professionals, overheads, and depreciation) were estimated by combining local reports, peer-reviewed literature, and personal communication and allocated per patient based on patient volumes. Only one facility provides a minimal amount of PD, and thus there remain uncertainties regarding the PD unit costs. All costs are incurred during the six weeks of treatment; no additional costs of complications were included. For further details of costs included, see Appendix 3.

**Table 2:** Input parameters

Parameters	Base Case	Sensitivity analysis	Distribution	Sources	Source number
**Disease Burden**					
Prevalence	2.8%			Igiraneza et al. 2018	[19]
In-hospital annual mortality from not recovering from PD or HD	34%			Igiraneza et al. 2018	[19]
Annual mortality for AKI hospital survivors on dialysis from other comorbidities	8.2%	7.2%–9.2%	Normal	Klarenbach et al. 2009	[27]
Annual in-hospital mortality from PD or HD complication plus other comorbidities	63%	45%–65%	Normal	Klarenbach et al. 2009	[27]
**Unit costs per patient (RWF, inflated, 2022)**					
***Total costs***					
**Payer perspective (full 18 sessions)**					
Costs of PD treatment	3,187,259	± 30%	Gamma	RSSB 2018	[7]
Cost of HD treatment	3,656,194	± 30%	Gamma	RSSB 2018	[7]
**Payer perspective (5 sessions)**					
Costs of PD treatment	1,687,615	± 30%	Gamma	RSSB 2018	(7)
Cost of HD treatment	1,890,082	± 30%	Gamma	RSSB 2018	[7]
***Direct medical costs***					
Bundled cost of catheter, drugs, labs, etc. PD	955,589	± 30%	Gamma		
Bundled cost of catheter, drugs, labs, etc. HD	613,262	± 30%	Gamma		
Palliative care (same for PD and HD)	690,296	± 30%	Gamma	Afiatin et al. 2017	[11]
Kit costs HD	1,984,847	± 30%	Gamma		
Dialysate costs PD	1,474,494	± 30%	Gamma		
***Direct non-medical costs***					
Staff costs PD	530,024	± 30%	Gamma	HLMA 2019, Author’s calc	[30]
Overheads PD	227,153	± 30%	Gamma	Aboagye et al. 2010, Author's calc	[31]
Staff costs HD	716,146	± 30%	Gamma	HLMA 2016, RSSB 2018	[7, 30]
Overheads HD	306,920	± 30%	Gamma	Aboagye et al. 2010, Author's calc	[31]
Annualized machine depreciation HD	35,019	± 30%	Gamma	Authors’ assumption	
*Operating costs = *staff* + *overhead*					
**Transition probabilities**					
Transition probability HD complication to hospital PD	1%	0.5%–5%	Beta	Authors’ assumption	
Transition probability PD complication to hospital HD	1%	0.5%–5%	Beta	Authors’ assumption	
Transition probability hospital HD to HD complication	4%	2%–6%	Gamma	Afiatin et al. 2017	[11]
Transition probability hospital PD to PD complication	25%	20%–50%	Gamma	Afiatin et al. 2017	[11]
Transition probability of HD complication to partial recovery	0.2%	0.1%–0.5%	Beta	Authors’ assumption	
Transition probability of PD complication to partial recovery	0.2%	0.1%–0.5%	Beta	Authors’ assumption	
Transition probability HD complication to recovered	71%	46%–82%	Beta	Klarenbach et al. 2009	[27]
Transition probability PD complication to recovered	71%	46%–82%	Beta	Klarenbach et al. 2009	[27]
Transition probability HD complication plus other complications	0.2%	0.1%–0.5%	Beta	Authors’ assumption	
Transition probability PD complication plus other complications	0.2%	0.1%–0.5%	Beta	Authors’ assumption	
Transition probability hospital HD to not recovery	34%	50%–80%	Beta	Igiraneza et al. 2018	[19]
Transition probability hospital PD to not recovery	34%	40%–75%	Beta	Igiraneza et al. 2018	[19]
**Utility**					
Utility of dialysis independent	0.81	0.65 -0. 90	Normal	Garay et al. 2019	[28]
Utility for PD without complication	0.62	0.52 – 0.72	Beta	Klarenbach et al. 2009	[27]
Utility for HD without complication	0.62	0.52 – 0.72	Beta	Klarenbach et al. 2009	[27]
Utility for PD with complication	0.31	0.13 – 0.49	Beta	Afiati et al. 2017	[11]
Utility for HD with complication	0.37	0.15 – 0.59	Beta	Afiati et al. 2017	[11]
**Discounting**					
Discounting rate for cost	3%	0%–5%		iDSI Reference Case	[23]
Discounting rate for utility	3%	0%–5%		iDSI Reference Case	[23]
*HLMA: Health Labour Market Survey 2016; RLFS: Rwanda Labour Force Survey 2016*					

Costs in the model are expressed in RWF and are inflated to 2022 prices using the Consumer Price Index [26]. They are converted to current US dollars using the latest available exchange rate of 1 United States dollar (US$):1030 RWF [27].

### Effectiveness

Clinical effectiveness parameters were drawn from several sources. Population mortality rates were sourced from the World Health Organization’s Global Health Observatory Data repository[28]. Mortality rates for AKI patients undergoing dialysis were from a local observational study [21]. Transition probabilities were from an Indonesian study that compared HD and PD for patients with ESRD [14], and on assumptions made by co-authors of this study.

Quality-adjusted life years (QALYs) were the primary health outcome in this study. Utility values for dialysis patients with AKI were sourced from two studies from Argentina and Canada, which used the EuroQoL EQ-5D-3L [29, 30]. Utility values for dialysis independence was sourced from Garay et al. [30].

### Discounting

Costs and outcomes were discounted at a standard 3% per annum after the first year following the iDSI Reference Case, though most costs are incurred in the first year[25]. The impact of varying the discount rate between 0 to 5% was explored in a sensitivity analysis [31].

### Thresholds

The base case analysis uses a cost-effectiveness threshold of 0.5 × GDP per capita (RWF 444,074 or US$431). This is broadly in line with recently estimated values for Rwanda based on cross-country studies of US$325 to US$426 (2022), or 39%–51% of GDP per capita[32–34].

### Analyses

The cost-effectiveness analysis was completed in TreeAge software (version 2023 R1.2), and the budget impact analysis in Microsoft Excel. Uncertainty was analyzed using one-way sensitivity analysis and probabilistic sensitivity analysis (PSA), with distributions set according to standard practice for different parameter types.

## Results

### Base-case results

Overall, the intervention (PD) was less expensive and less effective relative to the comparator (HD). Table 3 presents the incremental costs and QALYs for the intervention (PD) and status quo (HD), stratified by age. The total estimated per patient cost for PD was RWF 1,824,886 (US$1,771) compared with a total estimated cost of HD of RWF 2,059,354 (US$1,999). The expected net QALYs lost in delivering PD compared with HD were -0.62 for patients aged 15–49 and -0.27 for those over 50, the latter due to the older cohort’s increased mortality rate.

**Table 3:** Incremental cost-effectiveness analysis

		Cost in RWF (2022)	Inc cost in RWF (2022)	Cost in US$ (2022)	Inc cost in US$ (2022)	Effect QALY	Inc effect	ICER in RWF (2022)	ICER in US$ (2022)
Payer Perspective (Age 15–49 years)	Intervention (PD)	1,824,886		1,771		10.01			
	Status quo (HD)	2,059,354	-234,468	1,999	-228	10.63	-0.62	378,174	367
Payer Perspective (Age > = 50 years)	Intervention (PD)	1,824,886		1,771		5.13			
	Status quo (HD)	2,059,354	-234,468	1,999	-228	5.4	-0.27	868,399	843

At a threshold of 0.5 × GDP per capita (RWF 444,074 or US$431), the analysis suggests that HD provision, as the standard of care, was cost-effective compared with PD provision for patients aged 15–49, with an ICER below the threshold at RWF 378,174 (US$367). Notably, the interpretation of the ICER (Table 3) is reversed, because incremental costs and effects are both negative[35]. In other words, the ICER falls below the threshold, and thus the *comparator* (HD) is considered cost-effective.

For patients above 50, the analysis suggests that PD was the preferred option compared with HD with an ICER of RWF 868,399 (US$843). The same reverse interpretation of the ICER also applies to this scenario; as the ICER is above the threshold, PD is the preferred option (Table 3).

### One-way sensitivity analysis

One-way deterministic sensitivity analysis was applied to individual parameters that affected the ICER most. Varying the costs of HD kits, HD commodities, HD salaries, and HD overhead by ± 30% increased the ICER when the cost of each parameter increased, and decreased the ICER when the cost of each parameter decreased. When varying the cost of PD dialysate, PD commodities, PD salaries, and PD overhead by ± 30%, increasing the costs of the parameters decreased the ICER, and decreasing the costs increased the ICER. In other words, this suggests that there may be opportunities to reduce HD-related costs and enhance the favorability of the ICER; but the same is not true for PD. See Appendix 4 for a tornado diagram.

### Scenario analysis

In the reduced provision scenario, HD appeared to be cost-effective compared with PD. Again, for both age groups, the ICER falls below the threshold (Table 4).

**Table 4:** Scenario analysis

		Cost in RWF (2022)	Inc cost in RWF (2022)	Cost in US$ (2022)	Inc cost in US$ (2022)	Effect QALY	Inc effect	ICER in RWF (2022)	ICER in US$ (2022)
Scenario analysis: 5 sessions (Age 15–49 years)	Intervention (PD)	1, 075,065		1,043		10.01			
	Status quo (HD)	1,176,297	-101,232	1,142	-98	10.63	-0.62	163,278	158
Scenario analysis: 5 sessions (Age > = 50 years)	Intervention (PD)	1,075,065		1,043		5.13			
	Status quo (HD)	1,176,297	-101,232	1,142	-98	5.4	-0.27	374,934	364

### Probabilistic sensitivity analysis

**Fig. 1:**
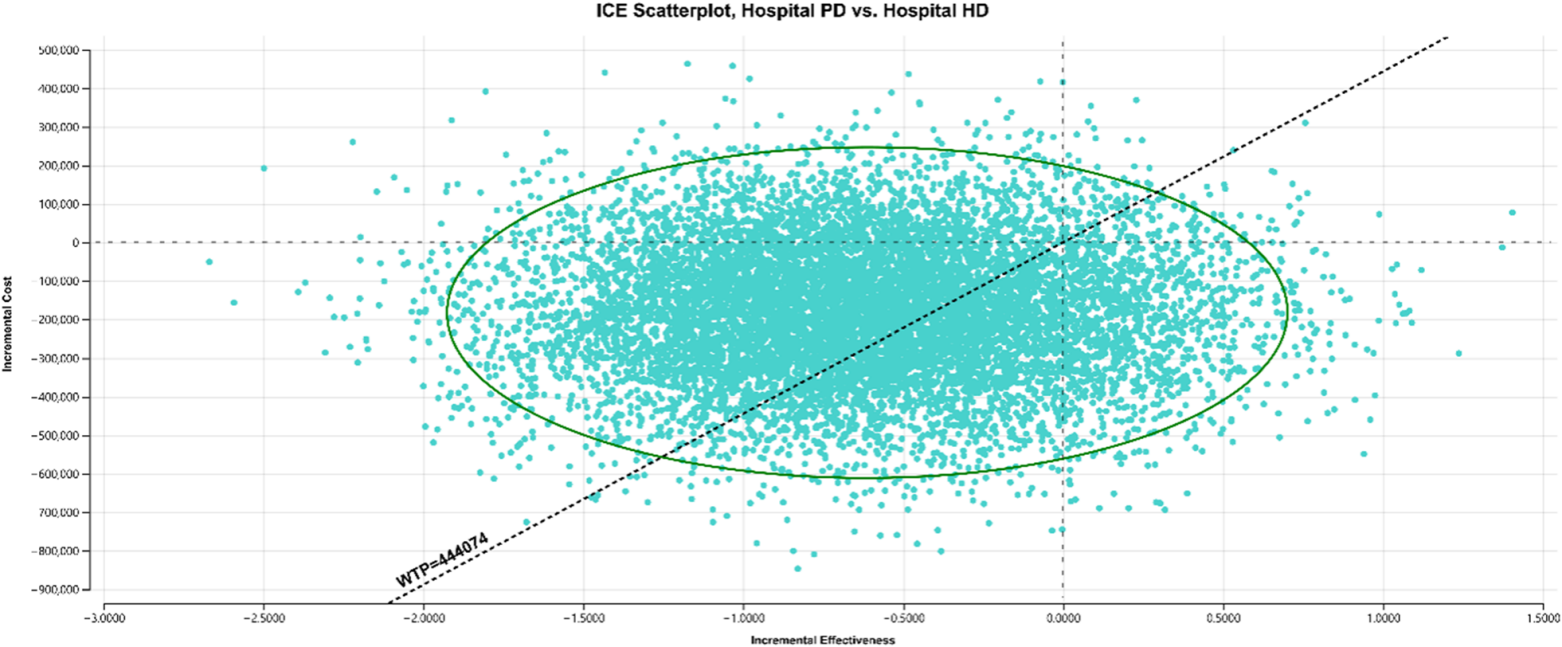
Incremental cost-effectiveness scatterplot

**Fig. 2:**
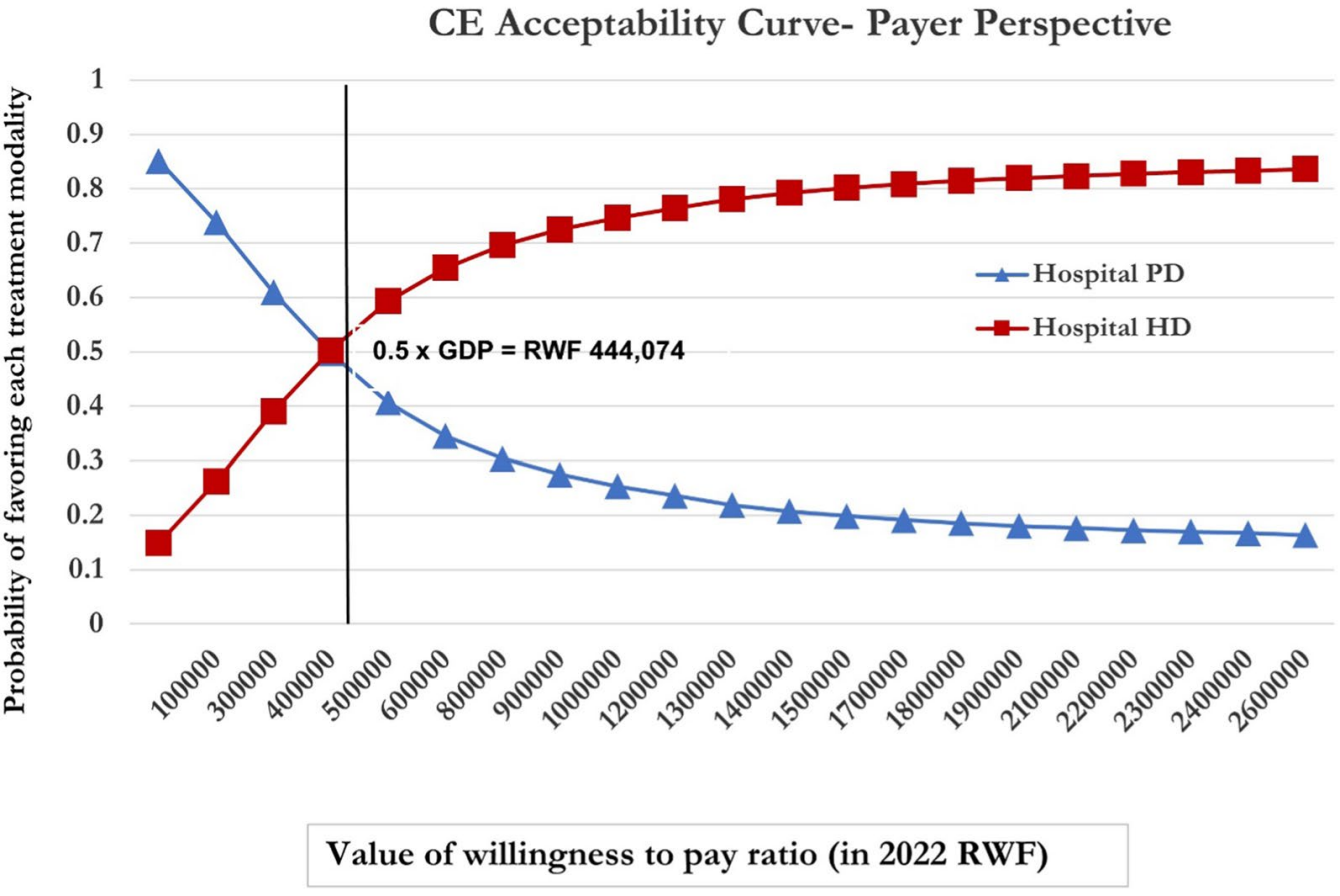
Cost-effectiveness acceptability curve

PSA was used to estimate the joint impact of uncertainty in all input parameters. Gamma distributions were applied to costs and beta distributions to health utilities (Table 2). By randomly sampling from each parameter distribution, 10,000 Monte Carlo simulations of incremental costs and incremental effects were obtained. The results of the PSA are presented in two figures. An incremental cost-effectiveness scatterplot (Fig. 1) and a cost-effectiveness acceptability curve (CEAC) (Fig. 2), which both summarize the impact of uncertainty in relation to the threshold[36]. At the threshold of 0.5 × GDP (RWF 444,074 or US$431) and above, HD provision has a 56% probability of being cost-effective relative to PD.

### Budget impact analysis

The budget impact analysis rapidly assessed the five-year (2020–2024) costs associated with providing dialysis services in four scenarios. In the baseline scenario, ‘HD preferred,’ a stable distribution of HD (91%) to PD (9%) was maintained (i.e., status quo). Alternative scenarios included scenario 1–5% annual shift to PD provision over 5 years; scenario 2–10% annual shift to PD provision over 5 years; and scenario 3—HD provision maintained at 91% but with reduced costs for HD kits. Compared to an ‘HD preferred’ baseline scenario, shifting to PD coverage would generate some savings. Maintaining an HD-preferred strategy and decreasing the cost of HD kits could achieve significantly more savings because HD kits represent more than half of the overall baseline cost of dialysis (Table 5).

**Table 5:** Budget impact results by scenario

	Baseline	Scenario 1	Scenario 2	Scenario 3
	HD preferred	5% Δ to PD	10% Δ to PD	HD + efficiency
5-year cumulative costs				
RWF	2,071,800,000	2,036,700,000	2,001,100,000	1,616,900,000
US$	2,000,000	2,000,000	1,900,000	1,600,000
Cost difference versus baseline scenario				
RWF		(35,100,000)	(70,700,000)	(454,900,000)
US$		-	(100,000)	(400,000)
Average cost per patient				
RWF	3,011,337	2,960,320	2,908,576	2,350,145
US$	2,907	2,873	2,762	2,326

## Discussion

At a threshold of 0.5 × GDP per capita, the analysis suggests that HD is cost-effective compared with PD for most AKI patients receiving dialysis in Rwanda, i.e., those aged 15–49 years. The budget impact analysis suggests that shifting to PD would cost less than maintaining the status quo over five years. However, it also suggests that maintaining the HD status quo *and* decreasing the cost of the HD kit, which is a major cost driver in providing HD, would save even more than shifting to PD. A reduction in the cost of the kit could also reduce the overall co-pay for the patient.

One-way sensitivity analysis suggests that decreasing the cost of HD commodities and kits decreases the ICER. This may be achievable, as one hospital, Rwanda Military Hospital (RMH), procures HD kits directly from a local supplier and pays about half the price of those procured for other hospitals providing dialysis [9]. If all hospitals were to get the RMH price for kits, the ICER would decrease to below the threshold for all ages, and HD would be more cost-effective relative to PD. Indeed, this information has led to ongoing price negotiations for the kits for all facilities.

This study contributes to a sparse literature on dialysis for AKI. A recent systematic review published after the time of analysis identified only seven other studies on dialysis for patients with AKI, with mixed results and a recent increase in industry-sponsored studies [37]. Our study is thus a valuable, independent contributor to this sparse literature.

From a cost-effectiveness perspective, this analysis should be seen only as a starting point for discussion rather than a policy recommendation. Indeed, results of the PSA illustrate that HD is slightly more cost-effective at the threshold compared with PD, but there is still uncertainty. Moreover, policymakers raise several issues that the analysis cannot address. These include the cost or requirements for changing or expanding services; the cost-effectiveness of service delivery at lower levels of care; and the impact of removing patient co-pays.

Additionally, since the time of analysis, dialysis policies have changed in Rwanda. More coverage of dialysis is now available, as is kidney transplantation. Our analysis reflects the coverage at the time of analysis, and further analyses could be conducted to reflect current available health services.

### Limitations

Our rapid cost-effectiveness analysis has several important limitations.

The study team’s approach to data collection was largely pragmatic, given time constraints. No attempts were made to synthesize the evidence for input parameters quantitatively or to systematically quality assure the data using available checklists. Data for the model came from several sources focused on patients with AKI where possible and supplemented by studies on CKD, author assumptions, and personal communication. Utility values, survival data, and transition probabilities are from various international sources. Importantly, the age of patients in papers from which utility values were sourced ranged from 45-to 65, while the average Rwandan dialysis patient is 38, and thus, utilities were overestimated. Local costing data were valuable in contextualizing the study. However, they were more focused on HD due to limited provision of PD, and they excluded the cost of infrastructure, overhead, and staff time for both HD or PD [9]. Other local reports, co-author assumptions and personal communication were used to fill data gaps, including staff time and equipment costs. Overhead was estimated as a percentage of operating costs (overhead + staff). Uncertainty remains about the costs and resources needed for PD because the local secondary data used reflected sparse provision of PD in hospitals. If time had allowed, the study would have benefited from more detailed costing on PD.

While the aim was to complete the analysis within six weeks, ultimately the assessment took about three to four months to complete.

### Generalizability

A few factors may limit this study’s generalizability. First, these results reflect an analysis of PD and HD delivered in a tertiary care setting due to current practice and data availability. If the intervention had been PD delivered at lower-level facilities or at home, as is often the case in other countries, the analysis may have found PD to be much more cost-effective[13]. Second, these results are based on a time and data-constrained analysis that pragmatically sourced local and international data. This increased the chance of uncertainty and bias. Our findings may have limited generalizability to other contexts and should not be interpreted without caution alongside other studies on dialysis in LMICs. Other studies often focus on ESRD patients who can either get HD in hospital or PD in lower-level facilities or at home, and often conclude that a PD-first policy is preferable[13–16]. If policymakers in Rwanda were considering coverage options for patients with ESRD or lower-level provision of PD, a separate cost-effectiveness analysis and budget impact analysis would need to be undertaken to understand the implications of the new policy choice.

### Reflections on ‘adaptive’ HTA

This cost-effectiveness analysis undertook an aHTA approach by deviating from what may be regarded as the ‘gold standard’ of HTA. This was done to reflect the local policymaker context, the availability of data, and general practicality constraints. The iDSI reference case served as a crucial principles-based framework to explore the suitability of the present analysis. Strategic choices were made on how to deliver evidence given the constraints, in a way that was still fit for policy makers’ purposes. This was done in two ways.

First, policymakers sought to conduct a rapid HTA to have 'proof of concept' for using HTA in decision-making. To conduct the analysis quickly, a topic was selected for which there were locally available cost data and supplemented by a pragmatic approach to collecting additional data as described in the limitations above. The implication of these choices naturally have impacts on the generalizability and potential bias of the analysis.

Second, the choice to exclude a 'no comparator' arm was made to reflect the local context. Dialysis is a hotly debated topic everywhere due to its high cost, and for ESRD, its limited effectiveness. From a purely economic perspective, some have argued that dialysis is an inefficient use of resources better spent elsewhere [38]. However, dialysis is a good illustration that priority-setting choices are not limited to cost-effectiveness. Dialysis is provided in many countries, including LMICs, on the grounds of financial risk protection and it being a moral imperative for universal health coverage [39]. The reality is that many LMICs have a shifting burden of disease, with existing coverage for dialysis services in LMICs being described as inadequate [40]. There is a real need to provide evidence to inform open debates about the optimal solution to providing dialysis.

Thus, the 'adaptive' choices in methodology made by the co-authors for this paper reflect a conscious effort to address policymaker needs in a deliberate departure from ‘gold standard’ HTA approaches. The pilot successfully raised awareness about HTA among key stakeholders, provided evidence for price negotiations, and identified key data needs that should be considered part of a strategy to support HTA development in the country [41, 42].

## Conclusion

Our de novo model suggests that HD may be cost-effective from the payer perspective compared with PD, and significant cost savings may be achieved by reducing the costs of HD commodities. While the relative robustness of this economic evaluation was constrained by adopting an aHTA approach, it was nonetheless a useful policy tool for Rwandan policymakers as it helped build the foundation for evidence-based priority setting in the future.

## Figures and Tables

**Fig. 3:**
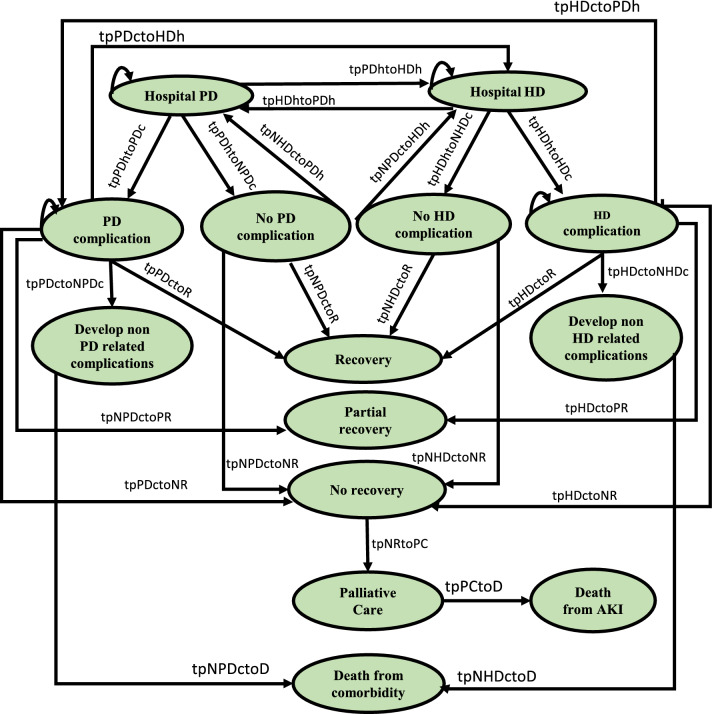
Markov Model Structure AKI eligible for PD vs. HD dialysis

**Fig. 4:**
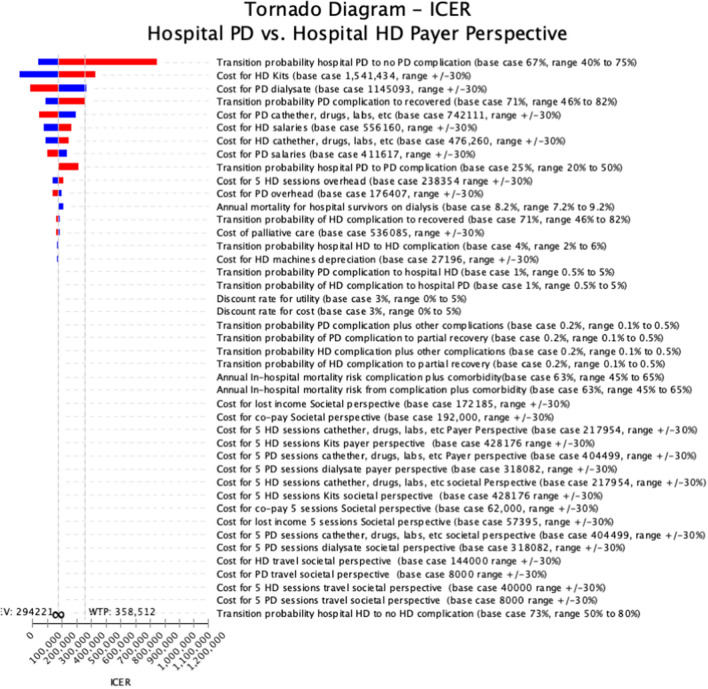
One-way sensitivity analysis

## Data Availability

The datasets used and/or analysed during the study are not publicly available due to the analysis being completed under the permission of RSSB, but are available from the corresponding author, in consultation with RSSB on reasonable request.

## Appendix 1.

See Fig. 3.

## Appendix 2: Data collection

To drive the search strategy, the team developed an initial *high level* data summary with needs and potential sources for the HTA—as presented in Table 6 below—and shared it with key policy makers.

**Table 6:** Data summary

Parameters	Potential sources
Disease burden	
Population	World Bank; Rwanda National Institute of Statistics
Prevalence	IHME; local observational studies
Mortality	WHO population life tables; demographic and heath survey (DHS); randomized control trials; local observational studies
Effectiveness	
Clinical effectiveness + quality of life	Systematic reviews of RCTs / RCTs; meta-analysis; systematic reviews; LMIC/LIC studies
Costs	
Costs of HD	Recently published cost analysis; national tariffs; personal communication
Costs of PD	Same as HD
Cost of palliative care	LMIC/LIC studies
Cost to patient	Patient surveys; hospital billing/claims; personal communication
Resource use data	
Resource Use (e.g. health staff time, facilities, consumables)	Prospective data collection/ local billing data; previous economic evaluations; personal communication

The initial data request was then expanded with iterative data collection based on the availability of local information and the willingness of local stakeholders to engage in the process and provide more detailed information. The search strategy was then based on a pragmatic approach, including the following steps:

1. The HTA developers reviewed an already completed iDSI review on dialysis requested by RSSB in early 2019 which summarized the available evidence on the costs and cost-effectiveness of dialysis for AKI patients with a focus on SSA and specifically, Rwanda.
2. Four local nephrologists and one international nephrologist expert on dialysis were identified. Consultation with these experts helped to unearth additional published and unpublished studies on clinical effectiveness and financing/costing studies in the field of dialysis of which experts had knowledge. These consultations also informed the structure of the model by clarifying the care pathway for patients with AKI.
3. Where there were data missing, the team drew on the iDSI network to source reliable dialysis models and studies. This included models and studies from Thailand’s Health Intervention and Technology Assessment Program (HITAP), a core partner of iDSI, which has led important dialysis studies in Southeast Asia and are known to represent a reliable source of information to support this model development.
4. A simple literature search strategy supplemented the above information. It was designed and used to search two electronic databases: EMBASE (Ovid) and PubMed (Ovid). This simple search strategy combined terms for the interventions or comparators of interest with terms for the target condition (dialysis for AKI) as well as terms for CKD as it was not the target condition, but the authors expected it to be better studied than AKI. It was also focused on available studies in Sub-Saharan and/or East Africa, to establish whether there are more locally relevant studies than those sourced through the above methods.

Full details of the terms used in the literature search are presented below in Table 7.

**Table 7:** Search criteria

1	Dialysis OR PD OR HD OR peritoneal dialysis OR haemodialysis OR hemodialysis
2	AND AKI OR Acute Kidney Injury OR CKD OR Kidney Failure OR Chronic Kidney Disease OR Kidney failure
3	AND Test OR testing OR treatment OR test*
4	AND Cost OR Costs OR Costing OR Cost-effectiveness OR Cost-effectiveness analysis OR effect* OR effective* OR expenditure OR healthcare costs
5	AND Africa OR SSA OR Sub-Saharan Africa OR Rwanda OR Kenya OR Uganda OR Tanzania OR Burundi OR Malawi OR Mozambique OR DRC OR Democratic Republic of Congo

Additional terms:

- Past 10 years only
- No limits applied for study design or language

## Appendix 3: Cost summary and breakdown

This appendix provides a detailed overview of costs included in each cost component and how they were calculated. All costs are expressed per patient and have been adjusted to 2022 RWF and current US dollars using the consumer price index (CPI) method (where adjusted costs = base year costs x (2022 CPI/base year CPI)), and the 2022 exchange rate US$ 1: RWF 1031

The Table 8 below provides an overview of costs which have been included in base case modelling, as well as the scenario

**Table 8:** Costs

	Payer perspective	Scenario
Sessions	18	5
PD Inputs	Direct medical costs; palliative care; dialysate; salaries; overhead	All costs @ 5 sessions
HD Inputs	Direct medical costs; palliative care; kits; salaries; overhead; depreciation	All costs @ 5 sessions

### Direct medical costs

Direct medical costs for PD included the cost of a PD catheter, catheter insertion, anesthesia, drugs, consumables, labs, dialysate, and palliative care. These figures were provided by RSSB as a supplement to the RSSB 2018 dialysis costing study; lab costs for PD and HD were assumed to be equivalent. Direct medical costs for HD included drugs, HD catheter, HD kit, laboratory tests, other acts/procedures (catheter insertion, IV injection, medical fee, and wound dressing), other consumables not specific to the dialysis kit, and other laboratory tests (those not done by all facilities). These figures were sourced directly from the RSSB 2018 dialysis costing study. Both interventions were presented in that study as a session cost for a ‘new’ case and a session cost for ‘old’ cases. For each, we summed the cost of the initial ‘new’ session plus a ‘per session’ cost for the remaining 17 sessions modelled (and in scenario analysis, the remaining four sessions). The per patient direct medical unit cost for HD was calculated using a straight line average of the total direct medical costs for provision across the four facilities where HD is delivered to CBHI patients (King Faisal Hospital (KFH), Rwanda Military Hospital (RMH), The University Teaching Hospital of Kigali (CHUK), and The University Teaching Hospital of Butare (CHUB)), while the cost for PD was sourced from the only hospital which provides PD services, KFH

### Direct non-medical costs

Both PD and HD incurred additional direct costs from salaries and overheads. Annual salary costs were calculated using a combination of staffing needs estimated by local experts and co-authors and salaries drawn from the 2019 Rwanda Health Labor Force Survey (which reported salaries in 2016 RWF). Staffing needs for PD and HD are summarized in Table 9 below. At CHUK, we assumed that 100% of each staff is allocated to HD services and used 2017–2018 patient volumes to calculate a unit cost per patient for staff. Then, due to uncertainty about staffing of PD services, we use the proportionate total cost of PD:HD services (74%) multiplied by the unit cost for HD services to estimate the unit cost for PD services (Table 9). Overheads were assumed to be 30% of total annual operating costs (staff + overheads)

**Table 9:** Staffing needs

Staffing type	Salaries (2022 RWF)	PD (KFH)	HD (CHUK)
Nurses	5,645,725	2	4
Nephrologists	22,363,115	1	1
General Practitioners	13,616,805	1	1
Lab Technicians	5,645,725	0	1
Cleaning staff	960,769	1	1
Total costs (2022)		48,232,139	65,169,314
PD: HD proportion		74%	
Volume of HD patients			91
Per patient HD staff cost		530,024*	716,146**

*Perpatient HD cost at CHUK * 74%

**Totalstaff costs at CHUK ÷ 91

## Appendix 4.

See Fig. 4

## Appendix 5.

See Table 10.

**Table 10:** CHEERS checklist

Section	Item No	CHEERS checklist—Items to include when reporting economic evaluations of health interventions	Yes/ Partly/ No/ Unclear/ NA	Page #
Title and abstract				
Title	Q1	Identify the study as an economic evaluation or use more specific terms such as “cost-effectiveness analysis,” and describe the interventions compared	Yes	p1, line 1
Abstract	Q2	Provide a structured summary of objectives, perspective, setting, methods (including study design and inputs), results (including base case and uncertainty analyses), and conclusions	Yes	p2, lines 4–23
Introduction				
Background and objectives	Q3	Provide an explicit statement of the broader context for the study. Present the study question and its relevance to health policy or practice decisions	Yes	p3 lines 15–22
Methods				
Target population and subgroups	Q4	Describe the characteristics of the base case population and subgroups analysed, including why they were chosen	Yes	p6 lines 3–4
Setting and location	Q5	State relevant aspects of the system(s) in which the decision(s) need(s) to be made	Yes	p6 line 3
Study perspective	Q6	Describe the perspective of the study and relate this to the costs being evaluated	Yes	p7 line 11–14
Comparators	Q7	Describe the interventions or strategies being compared and state why they were chosen	Yes	p6, line 6–9
Time horizon	Q8	State the time horizon(s) over which costs and consequences are being evaluated and say why appropriate	Yes	p6 line 1
Discount rate	Q9	Report the choice of discount rate(s) used for costs and outcomes and say why appropriate	Yes	p8 line 21–24
Choice of health outcomes	Q10	Describe what outcomes were used as the measure(s) of benefit in the evaluation and their relevance for the type of analysis performed	Yes	p8 line 17
Measurement of effectiveness	Q11a	Single study-based estimates: Describe fully the design features of the single effectiveness study and why the single study was a sufficient source of clinical effectiveness data	Yes	
	Q11b	Synthesis-based estimates: Describe fully the methods used for identification of included studies and synthesis of clinical effectiveness data		p8 line 12–16
Measurement and valuation of preference-based outcomes	Q12	If applicable, describe the population and methods used to elicit preferences for outcomes	Yes	N/A
Estimating resources and costs	Q13a	Single study-based economic evaluation: Describe approaches used to estimate resource use associated with alternative interventions. Describe primary or secondary research methods for valuing each resource item regarding its unit cost. Describe any adjustments made to approximate to opportunity costs	Yes	p7 line 20- p8 line 7
	Q13b	Model-based economic evaluation: Describe approaches and data sources used to estimate resource use associated with model health states. Describe primary or secondary research methods for valuing each resource item regarding its unit cost. Describe any adjustments made to approximate to opportunity costs		
Currency, price date, and conversion	Q14	Report the dates of the estimated resource quantities and unit costs. Describe methods for adjusting estimated unit costs to the year of reported costs if necessary. Describe methods for converting costs into a common currency base and the exchange rate	Yes	p8 line 8–10
Choice of model	Q15	Describe and give reasons for the specific type of decision-analytical model used. Providing a figure to show the model structure is strongly recommended	Yes	p7 line 2–3
Assumptions	Q16	Describe all structural or other assumptions underpinning the decision-analytical model	Yes	p7 line 5–9
Analytical method	Q17	Describe all analytical methods supporting the evaluation. This could include methods for dealing with skewed, missing, or censored data; extrapolation methods; methods for pooling data; approaches to validate or make adjustments (such as half-cycle corrections) to a model; and methods for handling population heterogeneity and uncertainty	Yes	p7 line 17–19
Results				
Study parameters	Q18	Report the values, ranges, references, and, if used, probability distributions for all parameters. Report reasons or sources for distributions used to represent uncertainty where appropriate. Providing a table to show the input values is strongly recommended	Yes	Table 2
Incremental costs and outcomes	Q19	For each intervention, the report means values for the main categories of estimated costs and outcomes of interest, as well as mean differences between the comparator groups. If applicable, report incremental cost-effectiveness ratios	Yes	Table 3
Characterizing uncertainty	Q20a	Single study-based economic evaluation: Describe the effects of sampling uncertainty for the estimated incremental cost and incremental effectiveness parameters, together with the impact of methodological assumptions (such as discount rate, study perspective)	Yes	
	b	Model-based economic evaluation: Describe the effects on the results of uncertainty for all input parameters, and uncertainty related to the structure of the model and assumptions	Yes	p11 lines 7–14, p12 lines 4–10
Characterizing heterogeneity	Q21	If applicable, report differences in costs, outcomes, or cost-effectiveness that can be explained by variations between subgroups of patients with different baseline characteristics or other observed variability in effects that are not reducible by more information	Yes	p10 lines 13–18
Conclusion				
Study findings, limitations, generalizability, and current knowledge	Q22	Summarize key study findings and describe how they support the conclusions reached. Discuss limitations and the generalizability of the findings and how the findings fit with current knowledge	Yes	p14 lines 4–13, p15 lines 1–6
Source of funding	Q23	Describe how the study was funded and the role of the funder in the identification, design, conduct, and reporting of the analysis. Describe other non-monetary sources of support	Yes	p19 lines 17–18
Conflicts of interest	Q24	Describe any potential for conflict of interest of study contributors in accordance with journal policy. In the absence of journal policy, we recommend authors comply with the International Committee of Medical Journal Editors recommendations	Yes	p19 line 15

## Abbreviations

aHTA: Adaptive health technology assessment
AKI: Acute kidney injury
CBHI: Community-based health insurance
CKD: Chronic kidney disease
ESRD: End-stage renal disease
GDP: Gross domestic product
HD: Hemodialysis
HTA: Health technology assessment
ICER: Incremental cost-effectiveness ratio
iDSI: International decision support initiative
LMIC: Low- and middle-income country
PD: Peritoneal dialysis
PSA: Probabilistic sensitivity analysis
QALY: Quality-adjusted life year
RMH: Rwanda Military Hospital
RSSB: Rwanda Social Security Board
RWF: Rwandan franc
US$: United States dollar
